# Puerperal Convulsions

**Published:** 1855-08

**Authors:** T. D. Washburn

**Affiliations:** Lawrenceville, Ill.


THE NORTH-WESTERN

MEDICAL AND SURGICAL JOURNAL.

NEW SERIES.

VOL. IV. AUGUST, 1855. NO. 8.

### ORIGINAL COMMUNICATIONS.

ARTICLE I.—*Puerperal Convulsions.* A paper read before the Esculapian Society at the semi-annual meeting, in May, 1855. By T. D. Washburn, M. D., of Lawrenceville, Ill.

On the 6th of March I was called to see Mrs. H., a young woman, perhaps, 19 years of age, of nervous-sanguine temperament, French extraction, dark complexion, eyes and hair; of moderate stature, with hereditary tendency to nervous debility and irritation. Previous to marriage, some two years since, she enjoyed average health, since which time, till she became *enciente*, she suffered from menorrhagia and became quite anæmic, and since conception she has been no better, and for the last two months has experienced considerable palpitation, with smothering sensations. Two days previous, while sitting in her chair, she fainted, and was compelled to lie down; about the same time she had some uterine pain, accompanied soon after with some "show," which has continued to the present time.

This morning, 8 o'clock A. M., I find her free from excitement, with moderate pains at intervals, and am informed she has gone her full time; bowels regular, and lower extremities much swollen. I left as a placebo, more than anything else, spirits lavender A., and curuph tinct. opii. A. A., to be given *pro a rata.*

Seven o'clock, P. M.—I called and found labor still progressing; os uteri dilated the size of a half dollar and relaxing kindly, but a close, snug build to contend with, and the parts not very well lubricated.

After an absence of a few hours, I was again at the bedside. The pains were short, but constant, with regular intervals; pulse uniform, and prospects favorable; natural presentation of the vertex. Toward morning the pains lulled, and as she was quite fatigued from loss of sleep, I gave her 20 drops of laudanum, and retired to recure sound rest for myself.

March 7th, 8 A. M.—I find my patient much the same as when I left. Pains moderate, but regular and gradually increasing; in the course of an hour the head became engaged, and made some advance, with the increase of the pains. Soon after nine o'clock the waters were discharged, but the pains did not become severe, though I remember about this time she exclaimed, "Oh! how my head does ache," still it did not excite my attention, as she was more or less subjected to it.

Her pains remained short, but became somewhat more vigorous, and, finally, with great difficulty the head was born; several more pains intervened before the child was delivered, which was a daughter, of ordinary size.

The placenta was expelled in ordinary time, with less than usual hemorrhage. The womb contracted well, and after applying a snug bandage, a little before noon she expressed herself as quite comfortable, with some disposition to sleep.

I retired soon after, and did not visit her again till past seven in the evening, when I found her friends in a state of extreme extreme excitement, saying they "thought her dying only a few minutes before," and had dispatched a messenger for me. On inquiry, I learned that she seemed quite easy till five o'clock, when she complained of her head and back, (her back had been the chief point of pain during her whole labor). At six o'clock, however, she partook freely of refreshment, and drank two cups of coffee, and was apparently doing well; soon after, she had a smothering paroxysm and became faint, but rallied directly, till just now she was taken worse, became unconscious, and they thought her dying. I find her now quite rational, rather slow of utterance, expression good, no unusual hemorrhage, the womb contracting well, and she complains only of moderate pain in her head and back. Ordered twenty drops of laudanum and left, expecting to return before bed time, but anticipating no serious difficulty.

I was absent some thirty minutes, when I was again called in great haste, and learned she was suffering from strong convulsions, but on arriving found her breathing natural, mind clear, but seemingly fatigued. It was not long, however, before all doubts were dissipated, for after becoming somewhat restless and sighing once or twice, she was seized with a most violent convulsion; every muscle of the body was brought into excessive action, her face was purple and distorted, her eyes rolled up or twitching, with saliva frothing from the mouth, and every symptom of the worst form of eclamsia, which continued for some five minutes or more, when she relapsed into stertorous breathing and slowly regained her consciousness, but rather more stupid than before.

I immediately directed strong sinapisms to the ankles, wrists and chest, with cold applications to the head; a short interval elapsed, when another convulsion came on more violent than before, with a slower return to consciousness, the pulse running up to 140 and gradually subsiding to 100. The mustard gave her considerable annoyance, besides, I directed enema of salt water, But all seemed to be useless, as the fits continued to increase, both in severity and frequency, and she became less and less conscious in the intervals.

Ten o'clock P. M., I apprised the husband of the nature and character of the attack, and the necessity of resorting to the most vigorous plan of treatment to accomplish any good, notwithstanding her delicate health and feeble condition, and that even the most energetic treatment might fail, and further suggested that another physician be called in if he wished my views corroborated. Dr. P. was sent for, and arrived directly. We at once decided to bleed, and opened a vein in her arm, making a fine incision, but the blood flowed thick and black, till some twelve or fourteen ounces had been discharged, when it flowed more freely with a more arterial hue, and seeming to give temporary relief to the patient. We also had her thick tresses of hair removed, to apply the cold effusion more effectually.

But a short time elapsed before the convulsions began with renewed vigor. The sinapisms, clysters and cold were faithfully applied, but the skin remained hot, and pulse accelerated; in the meantime, considerable flooding ensued, and about 2 o'clock in the morning she passed from stertorous into a less labored but restless sleep, her eyes remaining rolled far back, and perfectly unconscious to sound, light or taste, and unable to swallow. She continued thus for some five or six hours.

March 7th, 7 o'clock A. M.—After a little rest, I visited my patient, and found her nearly the same as when I left. Still sleeps almost natural, with eyes rolled upward, pupils much contracted, and entirely unconscious. The enemas had produced no effect. I now combined two ounces sulph. magnes. with two grains tart. emetic, in a tumbler of water, and succeeded in giving her two table-spoonfuls, and directed the nurse to continue the same every hour till some effect was produced, also hot fomentations to the vulva, and repeat the salt water clysters, with turpentine, every half hour, and sinapisms to the whole course of the spine.

As she became more rational, and began to yawn, and give indications of returning consciousness, she was again attacked between 8 and 9 o'clock with convulsions, as terrific as ever, and to all appearance, baffling all treatment; after a succession of five or six, about ten o'clock she had a copious evacuation, and toward noon sunk into a labored sleep, exhausted from the repeated convulsions; during the night previous, I should have mentioned, the lochia became much diminished, but the womb was in position, and the abdomen natural. At 3 o'clock, P. M., I found the convulsions making their appearance again, and despairing of arresting them with all the forces I could bring to bear, I opened a vein and took about a pound more of blood, so that the pulse became almost imperceptible.

During the succeeding two hours, she had three severe convulsions, and then sank into a profound sleep. In the course of the day, they had succeeded in giving most of the solution I had prepared in the morning, but it occasioned no nausea, though I think it assisted in checking the febrile excitement. At 6 o'clock P. M., she drank half a cup of tea without much difficulty, but seemed quite unconscious.

At 10 o'clock in the evening I succeeded in partially arousing her, so that when I spoke loudly she opened her eyes, but evidently could not see. She was now in a warm moisture, and the pulse had sank to 85.

I ordered 1–12 gr. ant. and 8 drops of laudanum, to be given every hour during the night

March 8th, A. M. at 7 o'clock—I visited Mrs. H. and found her much improved; more intelligence, calls for water, and says yes and no to queries; skin moist, pulse 80, uniform but feeble; tongue only slightly coated; complains of some pain in the head; has had another good evacuation from the bowels; some lochia.

℞—Very thin gruel, well cooked, half a tea-cupful every 3 hours; suspend other medicines, and take infus. valerian, a table-spoonful every two hours. Prognosis more favorable.

Did not see her again till six o'clock in the evening, she had been quiet during the day; complains of soreness and weakness, but is much more conscious, though not disposed to talk; relishes gruel and has some thirst, but is free from any general febrile excitements.

Ordered ether, c. spirits lavender and laudanum, equal parts, fifteen drops to be given every hour till she sleeps, and continue nourishment. At 1 o'clock at night I was suddenly roused by the husband of my patient, with the information that she was quite wakeful and very talkative, making many inquiries in regard to her sickness, of which she had no recollection. I gave him laudanum, with direction to continue ten drops every hour, till she obtained rest.

March 9th—This morning I find her quite natural: pulse 70, soft, skin moist, and expresses herself as much better, but sore all over. I directed her diet to be changed to rice gruel and tea. At 10 A. M., I called and found her quite feeble, the mere effort of raising the head to drink causing fainting, and, as she expresses it, "such a smothering feeling". I directed a tea-spoonful of wine in water every hour, in addition to her diet, and darken the room, and remove all noise.

P. M.—She seems to be doing well; has nursed her babe three times to-day; no action of the bowels. Directed the laudanum, as the night previous, to be followed with oil in the morning, if necesary.

March 10th.—Says she feels a "thousand times better" this morning; is perfectly intelligent and more composed; has rested well, taken the oil, and wishes more to eat; still complains of some pain in the back part of her head, and occasional numbness about her arms, which I advised to be rubbed with brandy and mustard or pepper; give the wine in the form of panada, and increase the quantity; expression good, tongue clean, baby well. Directed no medicine.

P. M., 7 o clock—No action from the medicine, but suffers no uneasiness. Give injections and repeat till the bowels are opened.

March 11th.—Continues better; bowels were moderately moved; plenty of secretion in the breasts, and all the functions seem to be discharging their duty. She has just ascertained the loss of her hair, and intimates that she will whip me for that piece of mischief. Continue light diet, occasionally a little broth, and use Seidlitz powders to keep the bowels soluble.

March 12th.—Still improving, and gains strength,—withdraw the wine; has some cramping in limbs,—continue low diet, and use cream of tartar *ad libitum.*

March 13th.—Has had some fever, but yielded promptly to antimony, laudanum and spirits of mindereri.

March 14th.—Doing well and discharged.

Subsequently, this patient was most too tenderly cared for, and she had a slow, tedious convalescence Both breasts inflamed and abscesses formed, which I lanced, and she slowly recovered.

This is the second case of the kind which has fallen to my lot to attend, during a residence of seven years in Illinois. Nor have I heard of but one other in the vicinity of my practice during that time.

My first patient was confined with her first child, but was quite advanced between thirty-five and forty years of age. The treatment in this first case was not as prompt, though equally and even more efficient, as *v. s.*, from the temporal artery, the use of excessive cold, chloroform, &c., with every variety of antiphlogistic treatment. She had more constitution than the latter, but the shock seemed to have been so great to the nervous system as to defy every attempt at adjustment again. Both gave birth to healthy children, and the attack in each was subsequent to parturition. The latter case impresses my mind, with overwhelming force, that in the whole catalogue of disease none demands such *prompt* and *daring* treatment—no disease admits of such powerful depleting remedies, and efficient antiphlogistic action, as this terrific and formidable malady, *puerperal convulsions.*

